# Support vector machine classification of patients with depression based on resting-state electroencephalography

**DOI:** 10.2478/abm-2024-0029

**Published:** 2024-10-31

**Authors:** Chia-Yen Yang, Yin-Zhen Chen

**Affiliations:** Department of Biomedical Engineering, Chung Yuan Christian University, Taoyuan 320314, Taiwan; Institute of Neuroscience, National Yang Ming Chiao Tung University, Taipei 112304, Taiwan

**Keywords:** depressive disorder, electrocorticography, entropy, power, support vector machine

## Abstract

**Background:**

Depression is one of the most common mental disorders. Although depression is typically diagnosed by identifying specific symptoms and through history, no recognized standard for depression diagnosis exists. This assures the development of objective diagnostic tools for depression.

**Objectives:**

We investigated the differences in the resting-state electroencephalograms (EEGs) of patients with depression and healthy controls (HCs) to distinguish patients from HCs by using a support vector machine (SVM) classifier with the following two feature selection approaches: t test and receiver operating characteristic analysis.

**Methods:**

We used the EEG data from the Patient Repository of EEG Data + Computational Tools; this study included 21 patients with depressive disorder (MDD) and 21 HCs. The relative frequency power, alpha interhemispheric asymmetry, left–right coherence, strength, clustering coefficient (CC), shortest path length, sample entropy (SampEn), multiscale entropy (MSE), and detrended fluctuation analysis (DFA) data were extracted to determine candidate EEG features associated with depression.

**Results:**

With the t-test selection, the SVM classifier demonstrated the highest performance with the accuracy, sensitivity, and specificity of 96.66%, 95.93%, and 97.550% for the eye-open condition and 91.33%, 90.59%, and 91.81% for the eye-closed condition, respectively. For comparisons of features in the 2 selection approaches, the most influential features were relative frequency power and left–right coherence.

**Conclusion:**

Using this information to distinguish patients with MDD from HC subjects with the SVM classifier resulted in a mean accuracy over 90%. Although this result may not be robust enough for clinical applications, further exploration is necessary given the simplicity, objectivity, and efficiency of the classifier.

Depression—the second most common mental disease of the 21st century—negatively affects more than 264 million people worldwide. It is characterized by a persistent feeling of sadness or a lack of interest in previously enjoyable activities. People with depression may also experience sleeping problems (insomnia or hypersomnia), purposeless physical activity (e.g., inability to sit still), difficulty in concentration or decision-making, improper movement proficiency (e.g., confusion about using the left or right hand), and slow speech [1]. After a long period, people may increase alcohol abuse, struggle in social interactions with others, and finally see suicide as a way out [2].

Currently, no efficient test is available for depression diagnosis. Depression diagnosis is typically based on the identification of specific symptoms and the history of depression [3]. However, the results of these behavioral observations and patient-reported tests (e.g., Diagnostic and Statistical Manual of Mental Disorders criteria [4] and Beck Depression Inventory [BDI]-II [5]) are solely based on the evaluator and patient judgments; thus, subjectivity is difficult to prevent in the results. Moreover, the signs required for diagnosis must be obvious or detectable. However, these signs could worsen if the condition progresses without timely diagnosis. Therefore, researchers have focused on developing objective diagnostic tools to help diagnose depression. For early intervention or treatment, an automatic auxiliary diagnostic tool with quick and easy-to-use features is crucial for the clinical assessment of depression.

In the past decade, research has demonstrated that patients with a depressive disorder have neurocognitive deficits [6]. The interest in evaluating the main differences between healthy people and patients with depression through studies, such as in resting-state functional magnetic resonance imaging and electroencephalogram (EEG) studies, is growing. For instance, Greicius et al. [7] examined resting-state functional connectivity in 28 people with major depressive disorder (MDD) and 20 healthy controls (HCs) to explore the effects of depression refractoriness. Their findings demonstrated that people with MDD exhibit abnormally increased thalamic and subgenual cingulate activity, suggesting that quantitative measures can be used to guide the therapeutic course. Moreover, Shim et al. [8] recorded resting-state EEGs in 87 patients with MDD and 58 HCs and analyzed the source-level network in global indices (i.e., strength, clustering coefficient [CC], path length, and efficiency) and nodal indices (i.e., eigenvector centrality and nodal CC). Their results showed that the disturbances in the network indices in people with MDD might reflect altered emotional processing, and that these indices might be useful MDD biomarkers.

With the advent of several computer-aided technologies, machine learning (ML) have become one of the major techniques applied in medicine. For instance, Ahmadlou et al. [9] used the wavelet-chaos methodology, Katz’s fractal dimension, and Higuchi’s fractal dimension (HFD) to investigate the non-linearity and complexity in the frontal lobe of patients with MDD. The resting eyes-closed (CE) EEG data from 12 adults with MDD and 12 adults with non-MDD demonstrated that the HFD of the beta band is more discriminative than that of the gamma band for discriminating between people with MDD and HCs. High accuracy (91.3%) was achieved for the classification of MDD and non-MDD EEGs. Hosseinifard et al. [10] also studied the non-linear features of EEG signals, including detrended fluctuation analysis (DFA), HFD, correlation dimension, and Lyapunov exponent, to discriminate 45 patients with depression from 45 HCs. Of the k-nearest neighbor, linear discriminant analysis, and logistic regression (LR) classifiers, the highest classification accuracy (90%) was achieved by all features and LR classifiers. Furthermore, Mumtaz et al. [11] found synchronization likelihood features to be better than interhemispheric coherence and mutual information for automatic MDD diagnosis. Classification models, such as support vector machine (SVM), LR, and naïve Bayesian, were employed to analyze EEG data from 34 patients with MDD and 30 HCs, and they showed high discrimination accuracy, indicating that the selection of a minimum number of features could simplify the model and thus reduce the computation costs in clinical applications.

Biological data are generally high-dimensional data and pose a large challenge for ML application. Hence, the feature selection is a key part of applied ML, which refers to the process of obtaining a small set of features containing the important data [12]. In this study, we investigated the differences in resting-state EEGs between patients with depression and HCs, with the aim of distinguishing the patients from the HCs by using a SVM classifier with the following two feature selection approaches: two-sample Student’s *t*-test and receiver operating characteristic analysis. To identify the candidate EEG features associated with depression, we evaluated various traditional and novel characteristics of brain activity (i.e., relative frequency power, alpha interhemispheric asymmetry, left–right coherence, strength, CC, shortest path length [L], sample entropy [SampEn], multiscale entropy [MSE], and DFA). After their identification, the significant characteristics were input into the SVM for iterative training and testing to construct a model for distinguishing patients with depression from HCs.

## Methods

### Subjects

In this study, EEG data were downloaded from the Patient Repository of EEG Data + Computational Tools (http://predict.cs.unm.edu/), an open dataset provided by Cavanagh et al. [13]. In total, 21 patients with MDD and 21 HCs (mean ± standard deviation age = 18.86 ± 1.35 years and 18.67 ± 0.73 years, respectively, *P* = 0.573) were included. For this dataset, individuals were recruited from introductory psychology classes based on mass survey scores on the BDI. They were all aged between 18 years and 25 years and did not have a past history of head trauma or seizures and a current history of psychoactive medication use. The grouping criterion was a score of >13 (mean ± standard deviation = 21.52 ± 5.66) and <7 points (1.00 ± 1.05) on the BDI for patients with MDD and HCs, respectively (*P* < 0.001). Additionally, patients with MDD underwent the Structured Clinical Interview for Depression and were categorized based on their interview outcomes: (1) those declining the interview, (2) those not meeting MDD criteria, (3) those meeting criteria for past MDD, and (4) those meeting criteria for current MDD. The selected participants with MDD met criteria for either past or current MDD. HCs were screened to ensure no self-reported history of MDD and no symptoms indicative of an Axis I disorder, assessed through computerized self-report completion of the Electronic Mini International Neuropsychological Interview.

To balance group sizes, we applied independent component analysis (ICA) to decompose data from 58 EEG channels into 40 components. Data with over 15 noisy components (e.g., eye movement, electromyography, or electrocardiography signals) were excluded. HCs were randomly selected with a uniform distribution to match the gender ratio and the age distribution of the MDD group.

### Data acquisition

During the experiments, all participants were instructed to reduce any movements and were asked to think of nothing for 5 min but without falling asleep. Electric potentials were recorded using whole-head 64 Ag-AgCl scalp electrodes on a Synamps 2 system (Neuroscan, Charlotte, NC, USA). Electrical responses were digitized at a sampling rate of 500 Hz and were recorded between 0.5 Hz and 100 Hz. EEG data were chosen from 58 channels according to the International 10–20 system. The online reference was a single-channel placed between Cz and CPz. The electrode impedance was <10 kΩ.

### Data analysis

All processing steps were executed using an in-house program written in MATLAB (MathWorks, Natick, MA, USA). EEG signals were preprocessed in the following three stages: first, all truncated signals were detrended to remove means, offsets, and slow linear drifts over the time course. Second, detrended signals were filtered using a 0.5–50-Hz band pass filter. Third, filtered signals were decomposed using the FastICA algorithm to manually remove components containing artifacts in the form of eye movements and electrocardiogram activity. After preprocessing, the cleaned signals were decomposed into the following five frequency bands through discrete wavelet decomposition: delta (0.5–4 Hz), theta (4–8 Hz), alpha (8–12 Hz), beta (12–30 Hz), and gamma (30–50 Hz). Seven signal features were then calculated with a 2-s non-overlapping window.

(1) Frequency power: this is the relative power calculated in each band, and it is divided by the sum of the power across the entire frequency.
(2) Alpha interhemispheric asymmetry: a total of 26 pair indices were calculated as (R − L)/(R + L), where R and L are right and left hemisphere powers, respectively.
(3) Left—right coherence: the signals of a total of 625 [=25 (left) × 25 (right)] electrode pairs were calculated for interhemispheric coherence.
(4) Structural properties of the network: coherence values obtained between electrodes over the entire brain were binarized into a network using a threshold, and then, its strength, CC, and L were calculated.
(5) SampEn: template vectors, Y_m_(i), constructed using a given embedding dimension *m* were used to measure the negative natural logarithm of the probability ln(ψ_m_(r)/ψ_m+1_(r)), where ψ_m_(r) denotes the number of template vectors having a distance, d(Y_m_(i), Y_m_(j)) = max|x_i+k_ – x_j+k_|, smaller than tolerance *r*.
(6) MSE: the coarse-grained time series generated by averaging different numbers of consecutive points was calculated using SampEn, and all these values were then summated.
(7) DFA: a cumulative sum, Y(i), calculated from the time-series dataset x, where i = 0, 1, …, N, was divided into time windows of length n and was linearly fitted using least-squared errors. The slope of the log of the root-mean-square deviation from the trend, F(n), against log n within each time window was then calculated using least-squares.

### Feature selection

All features were standardized using the z-score and randomly divided into two separate sets: 80% and 20% for training and testing datasets, respectively. The 2 types of selection methods were used for reducing the input dimensionality for improving the classification performance: (1) A two-sample Student’s *t*-test was used to compare the seven types of EEG features between the patients with MDD and HCs, with a statistical significance of *P* < 0.05. (2) The area under the receiver operator characteristic curve (AUC) of each feature was calculated according to the method of Mumtaz et al. [11], and absolute differences were ranked from high to low—where the closer the value was to 0.5, the better the discrimination was; whereas, a value of 0 corresponded to no discrimination (e.g., randomly flip a coin). For choosing features with high information, the number of features was increased from 1 to 1,000.

### SVM classification

After the feature selection, we used a SVM classifier with a radial basis function kernel to train and predict the results. The grid search and tenfold cross-validation were used to find the best hyperparameter, which were cost (*c*) and gamma (*g*), and to evaluate the performance of the trained model. The range of *c* and *g* was from 10^−10^ to 10^10^ with 10^0.2^ steps. Finally, the optimized model was tested using the testing dataset to evaluate the accuracy, sensitivity, and specificity of the model [14]. To verify the correctness of the optimized model, we shuffled the labels and conducted a permutation test. These processes were repeated 100 times.

## Results

### EEG features

**Figure 1.**
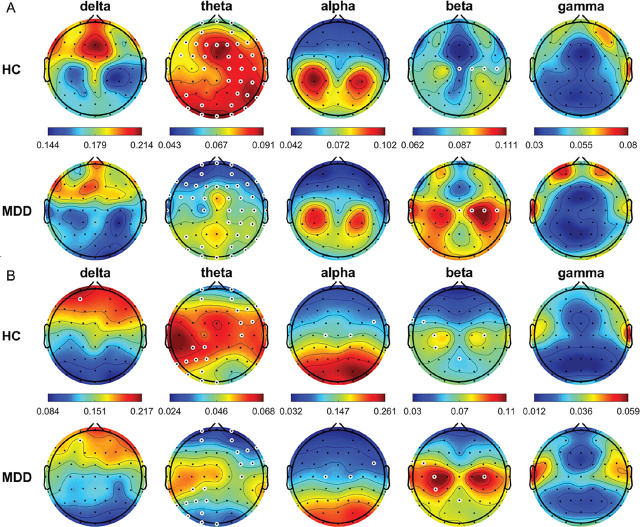
Average relative power of EEG signals (delta, 0.5–4 Hz; theta, 4–8 Hz; alpha, 8–13 Hz; beta, 13–30 Hz; and gamma, 30–50 Hz) from all HCs and patients with MDD in the OE (**A**) and CE (**B**) condition. White circles denote significant differences between groups (*P* < 0.05). CE, eyes-closed; EEG, electroencephalogram; HCs, healthy controls; MDD, major depressive disorder; OE, eyes-open.

The average relative power topographic maps for different frequency bands for all the HCs and patients with MDD are shown in **Figure 1**. In the eyes-open (OE) condition, theta power was significantly higher in the HCs than in the patients with MDD at FP1, FPz, FP2, AF3, AF4, F7, F5, F3, F1, FZ, F2, F4, F6, F8, FT7, FC5, FC3, FC1, FC2, FC4, FC6, FT8, T7, C1, C2, C4, C6, T8, CP2, CP4, CP6, TP8, P5, P4, P6, P8, PO7, PO3, PO4, PO8, O1, Oz, and O2 (all *P* < 0.05); whereas, beta power was significantly lower in the HCs than in the patients with MDD at T7, Cz, C2, C4, C6, and PO7 (all *P* < 0.05). In the CE condition, frequency power was significantly higher in the HCs than in the patients with MDD at AF3 in the delta band; FP1, FP2, AF3, AF4, F4, F6, F8, FC4, FC6, FT8, C4, CP3, CP1, P7, P5, P3, P1, PO7, PO3, O1, and Oz in the theta band; and FC6, C1, and C2 in the alpha band (all *P* < 0.05). By contrast, beta power was significantly lower in the HCs than in the patients with MDD at FT7, FC5, C3, C4, CP3, and Pz (all *P* < 0.05).

**Figure 2.**
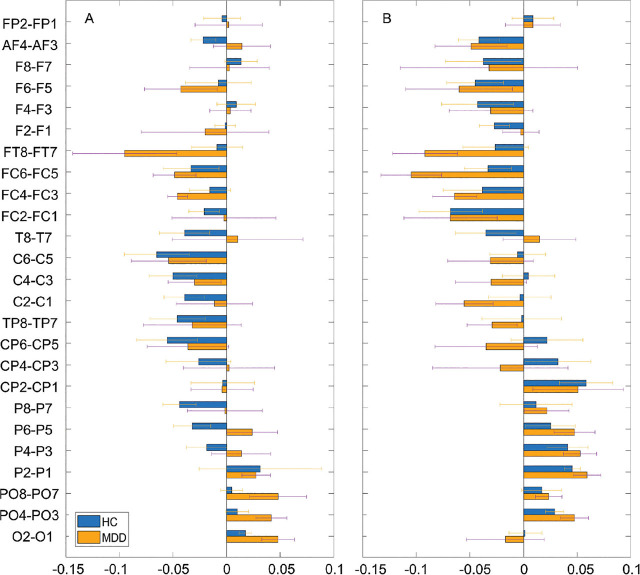
Average power asymmetry of relative activity between the pairs of electrodes (i.e., right location–left location) in HCs and patients with MDD in the OE (**A**) and CE (**B**) condition. HCs, healthy controls; MDD, major depressive disorder; CE, eyes-closed; OE, eyes-open.

**Figure 2** shows the asymmetry of a relative activity between the pairs of brain regions (i.e., right hemisphere–left hemisphere) across all subjects. In the OE condition, the values were negative around the central area in the HCs and around the frontocentral area in the patients with MDD. However, no significant differences were noted. In the CE condition, this tendency was more apparent. The values were more negative around the central and frontal areas in the HCs and around the frontocentral and central areas in the patients with MDD. In addition, the values were more positive around the occipital area in both the groups. However, no significant differences were found.

**Figure 3.**
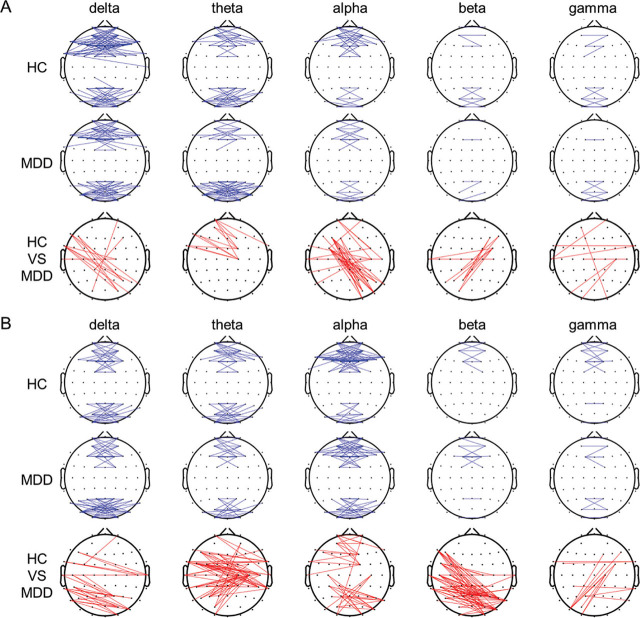
Grand functional connection maps of the left–right coherence between the pairs of electrodes at the threshold of 0.6 for different frequency bands during the OE **(A)** and CE **(B)** conditions. Red lines in the bottom panels denote significant differences between groups **(***P* < 0.05). CE, eyes-closed; OE, eyes-open.

**Figure 3** shows the connection maps of the left–right coherence between electrode pairs with significant differences between the HCs and patients with MDD. For easy comparison, we summed the numbers of pairs with significant differences within the following eight brain regions: left frontal, right frontal, left central, right central, left temporal, right temporal, left occipital, and right occipital [15]. In the OE condition, the values were higher in the HCs than in the patients with MDD for the theta and gamma bands but lower in the HCs than in the patients with MDD for the delta, alpha, and beta bands. The pairs that showed significant differences were right frontal–left occipital electrodes for the delta band, right frontal–left central electrodes for the theta band, right central–left occipital electrodes for the alpha band, right occipital–left central electrodes for the beta band, and right temporal–left temporal and right occipital–left central electrodes for the gamma band (all *P* < 0.05). In the CE condition, the values were higher in the HCs than in the patients with MDD for the theta and alpha bands but lower in the HCs than in the patients with MDD for the delta, beta, and gamma bands. The pairs that showed significant differences were right central–left occipital and right temporal–left occipital electrodes for the delta band, right central–left central electrodes for the theta band, right frontal–left frontal and right central–left occipital electrodes for the alpha band, right central–left occipital electrodes for the beta band, and right occipital–left central electrodes for the gamma band (all *P* < 0.05).

**Figure 4.**
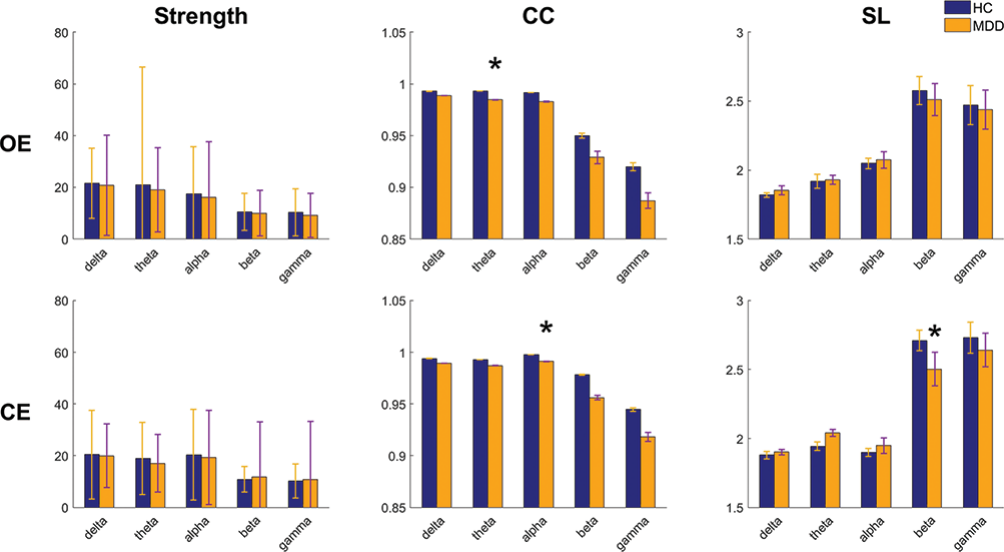
Grand average of parameters, namely, strength, CC, and L, calculated from the delta, theta, alpha, beta, and gamma bands during the OE and CE conditions. *indicates significant differences between groups (*P* < 0.05). CC, clustering coefficient; CE, eyes-closed; OE, eyes-open.

The mean strength, CC, and L of the constructed connection maps with an appropriate threshold in the OE and CE conditions are shown in **Figure 4**. In the OE condition, the values were higher in the HCs than in the patients with MDD for all the frequency bands, except for L. A significant difference (*P* = 0.026) was found for CC in the theta band. In the CE condition, a similar situation was noted. Significant differences were found for CC in the alpha band (*P* = 0.007) and for L in the beta band (*P* = 0.039).

**Figure 5.**
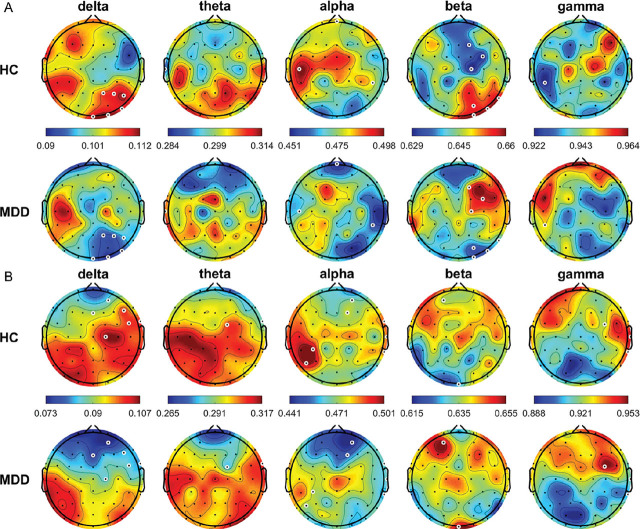
Average SampEn for the different frequency bands from all HCs and patients with MDD in the OE **(A)** and CE **(B)** conditions. White circles denote significant differences between groups (*P* < 0.05). CE, eyes-closed; HCs, healthy controls; MDD, major depressive disorder; OE, eyes-open; SampEn, sample entropy.

The SampEn was significantly higher in the HCs than in the patients with MDD at P2, P4, P6, PO8, Oz, and O2 in the delta band and FPz, C5, and CP6 in the alpha band (all *P* < 0.05) (**Figure 5**). By contrast, the SampEn was significantly lower in the HCs than in the patients with MDD at T7 in the theta band and F2, FC4, C2, P8, PO4, PO8, and O2 in the beta band (all *P* < 0.05). In the CE condition, the SampEn was significantly higher in the HCs than in the patients with MDD at AF4, Fz, F6, FC6, FT8, and C2 in the delta band and at AF4, F2, FT8, T8, CP3, TP8, and P5 in the alpha band (all *P* < 0.05). By contrast, the SampEn was significantly lower in the HCs than in the patients with MDD at AF3 and Oz in the beta band (all *P* < 0.05).

**Figure 6.**
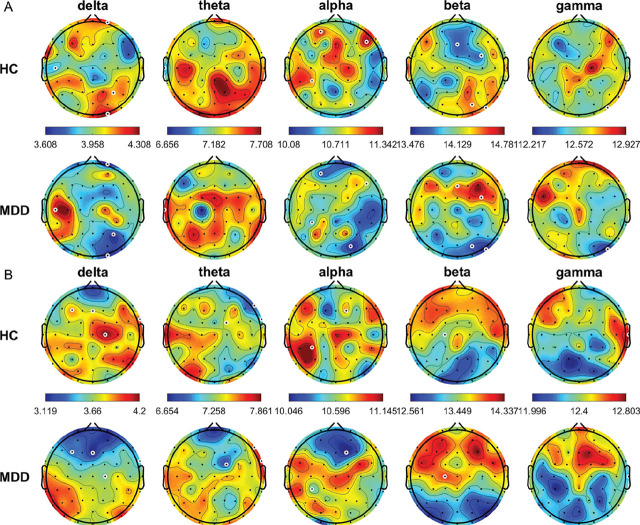
Average MSE for the different frequency bands from all HCs and patients with MDD in the OE **(A)** and CE **(B)** conditions. White circles denote significant between-group differences (*P* < 0.05). CE, eyes-closed; HCs, healthy controls; MDD, major depressive disorder; MSE, multiscale entropy; OE, eyes-open.

In the OE condition, the MSE was significantly higher in the HCs than in the patients with MDD at AF3, F6, CP3, and PO4 in the alpha band and PO8 in the gamma band (all *P* < 0.05) (**Figure 6**); whereas, the MSE was significantly lower in the HCs than in the patients with MDD at T7 in the theta band (all *P* < 0.05). In the CE condition, the MSE was significantly higher in the HCs than in the patients with MDD at F3, Fz, TP8, and C2 in the delta band; F2 and CP3 in the alpha band; and T8 in the gamma band (all *P* < 0.05).

**Figure 7.**
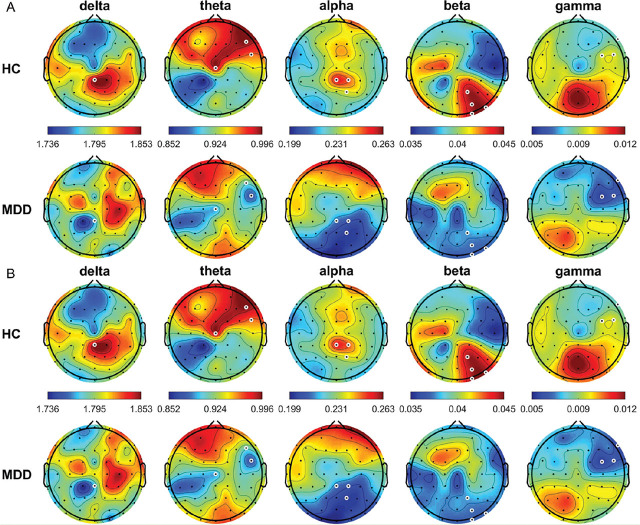
Average DFA for the different frequency bands from all HCs and patients with MDD in the OE (**A**) and CE (**B**) conditions. White circles denote significant differences between groups (*P* < 0.05). CE, eyes-closed; DFA, detrended fluctuation analysis; HCs, healthy controls; MDD, major depressive disorder; OE, eyes-open.

In the OE condition, the DFA was significantly higher in the HCs than in the patients with MDD at Cz in the alpha band, F7 in the beta band, and F3 and FC5 in the gamma band (all *P* < 0.05) (**Figure 7**); whereas, it was significantly lower in the HCs than in the patients with MDD at FC5, TP8, C5, and T8 in the delta band and P8 and O2 in the theta band (all *P* < 0.05). In the CE condition, the DFA was significantly lower in the HCs than in the patients with MDD at CPz in the delta band at F6, FC6, and Cz in the theta band; CPz, CP2, and P2 in the alpha band; P2, PO4, PO8, and O2 in the beta band; and F8, FC4, and FC6 in the gamma band (all *P* < 0.05).

### Feature selection

After feature extraction, 4,368 features were obtained. We used Student’s *t*-test to obtain 181 significant features in the eye-open condition (i.e., 49, 0, 88, 0, 1, 0, 18, 15, and 10 for the relative frequency power, alpha interhemispheric asymmetry, left–right coherence, strength, CC, L, SampEn, MSE, and DFA, respectively) and 293 significant features in the eye-closed condition (31, 0, 214, 0, 1, 1, 20, 12, and 14 for the relative frequency power, alpha interhemispheric asymmetry, left–right coherence, strength, CC, L, SampEn, MSE, and DFA, respectively). The three-time test accuracy of relative frequency power, alpha interhemispheric asymmetry, left–right coherence, structural properties of network, SampEn, MSE, and DFA in the SVM was 62.96%, 0%, 74.07%, 48.15%, 77.78%, 74.07%, and 66.67% in the eye-open condition, respectively, and 77.78%, 51.85%, 70.37%, 62.96%, 85.19%, 74.07%, and 51.85% in the eye-closed condition, respectively.

We also used AUC to select features by ranking their evaluation values. Since the accuracy is stabilized for the first 150 features, no additional features were added. According to the test accuracy in the three-time training of the SVM, the turning point at which the curve was over 95% and began to plateau was selected. In the eye-open and eye-closed conditions, the numbers of features were 17 (accuracy = 85.55%) and 9 (accuracy = 86.88%), respectively.

### SVM classification

After the feature selection, the features were input into the SVM classifier for training to obtain the classification model. The recognition accuracy was evaluated using tenfold cross-validation and was tested 100 times. In the eye-open condition, the accuracy, sensitivity, and specificity of the trained model (*c* = 0.004 and *g* = 0.047 after grid searches) for the *t*-test feature selection were 96.66% (CI = 1.132), 95.93% (CI = 1.666), and 97.550% (CI = 1.728), respectively (the permutation *P-*value was <0.001). The accuracy, sensitivity, and specificity of the trained model (*c* = 0.0009 and *g* = 0.0009 after grid searches) for the AUC feature selection were 85.55% (CI = 2.145), 87.76% (CI = 2.981), and 84.07% (CI = 3.673), respectively (the permutation *P-*value was <0.001). In the eye-closed condition, the accuracy, sensitivity, and specificity of the trained model (*c* = 0.0009 and *g* = 0.0009) for the *t*-test feature selection were 91.33% (CI = 1.785), 90.59% (CI = 2.885), and 91.81% (CI = 2.428), respectively (the permutation *P*-value was <0.001). The accuracy, sensitivity, and specificity of the trained model (*c* = 0.0001 and *g* = 0.1088) for the AUC feature selection were 86.88% (CI = 1.957), 87.73% (CI = 2.815), and 86.76% (CI = 3.402) (the permutation *P*-value was <0.001) (**Table 1**). Furthermore, in the eye-open condition, the total computation times for the *t*-test and the AUC feature selection were 76.42 and 276.61 s in the training process, respectively; whereas, both were 0.001 s in the testing process. In the eye-closed condition, the total computation times for the *t*-test and the AUC feature selection were 109.00 s and 265.44 s in the training process; whereas, both were 0.001 s in the testing process.

**Table 1.** Average accuracy, sensitivity, and specificity of the SVM models for the selected features

	**Number of features**	**Accuracy (%)**		**Sensitivity (%)**	**Specificity (%)**
		**Training**	**Testing**		
Eye-open	181	95.26 ± 3.30	96.66 ± 5.80	95.93 ± 8.54	97.55 ± 8.86
	17	84.99 ± 3.73	85.55 ± 10.99	87.76 ± 15.287	84.07 ± 18.83
Eye-closed	293	91.00 ± 3.97	90.77 ± 8.51	91.38 ± 13.06	89.11 ± 15.52
	9	87.65 ± 4.04	86.88 ± 10.03	87.73 ± 14.43	86.76 ± 17.44

SVM, support vector machine.

## Discussion

According to a review by Maitín et al. [16], SVM is the mostly used as an individual technique. To resolve the issue of high dimensionality in the dataset, the feature selection should be applied as a preprocessing step to improve the SVM classifier performance. Among several selection methods, filter-based approaches have low computational cost and high generalization capacity [17]. Thus, we constructed an SVM model to discriminate between patients with MDD and HCs after identifying discriminating features using two diverse feature selection approaches. The *t*-test selection approach demonstrated the highest performance in both the eye-open and eye-closed conditions—with an accuracy, sensitivity, and specificity of 96.66%, 95.93%, and 97.550% in the eye-open condition, respectively, and 91.33%, 90.59%, and 91.81% in the eye-closed condition, respectively. Notably, in the eye-open condition, the results were also significantly more favorable in the *t*-test selection than in the AUC selection, with significant differences (*P* = 0.0000, 0.0001, and 0.000 for accuracy, sensitivity, and specificity, respectively). In the eye-closed condition, the results were similar, but the difference was only significant for accuracy (*P* = 0.0035). The results were significantly more favorable in the eye-open condition than in the eye-closed condition for the *t*-test selection (*P* = 0.0000, 0.0066, and 0.000 for accuracy, sensitivity, and specificity, respectively). Moreover, the results were more favorable in the eye-closed condition than in the eye-open condition for the *t*-test selection, but the difference was non-significant (*P* > 0.05). In other words, although AUC could be used to select essential features, in a relative noisy condition, such as, when the eyes are open, using few features to train the classifier may not be sufficient to achieve a high performance [18]. By contrast, in a relatively stable condition, the selection approaches may not have considerable influence; thus, the next consideration was efficiency.

In a detailed comparison, we examined the features in the eye-open condition that achieved the highest performance. All the features selected using the AUC selection appeared in the *t*-test selection, including 7, 7, 1, and 2 features of relative frequency power, left—right coherence, SampEn, and MSE, respectively. In other words, regardless of the selection approach used, the most influential features were relative frequency power and left–right coherence. The relative frequency power analysis is a conventional approach that is used for finding early depression biomarkers, enabling the quantification of EEG features on a biological basis [19]. For instance, Nofzinger et al. [20] used electroencephalography and a positron emission tomography to clarify the neurobiological basis of the variations and demonstrated a similar relationship between electrophysiological arousal, including beta EEG power, and glucose metabolism in the ventromedial prefrontal cortex in people with depression and healthy individuals. In a review, Fernández-Palleiro et al. [21] concluded that the alterations in the alpha and theta rhythms could be neurophysiological markers of MDD risk, which may aid in the establishment of early diagnosis and treatment of mental state disorders applicable for the clinical practice in the future. In our study, the theta power exhibited the most obvious differences between the 2 groups. This finding attributed to diminished reward learning in the rostral anterior cingulate cortex (rACC) [22,23,24] or memory impairment caused by hippocampal volume reduction due to hormones or other protein molecules involved in depression [25]. Furthermore, the analysis of left–right coherence is a common approach to yield information on network formation and functional integration across brain regions [26]. For instance, Markovska-Simoska et al. [27] investigated the changes in the EEG coherence in groups of different mental disorders, such as depression, in the following 2 conditions: eyes opened and closed. The authors found the interhemispheric hyper coherence in people with depression, specifically for alpha and beta bands, and suggested that the EEG coherence analysis could be sensitive for the detection of abnormalities in patients with neuropsychiatric disorders. Moreover, McVoy et al. [28] investigated quantitative EEGs in 25 pediatric patients with MDD in comparison with 14 healthy children. Their results showed that in the frontal cortex, the patients with MDD had decreased resting connectivity in the alpha and theta bands, and they concluded that the impaired development of a resting-state brain network in the patients with MDD could be assessed using electroencephalography. Many studies have shown that abnormal interhemispheric communication in the human brain can result in sensory, motor, and language impairments observed in neuropsychiatric disorders, such as autism spectrum disorder, schizophrenia, and MDD [29,30,31,32,33]. The aberrant interhemispheric connectivity may be linked to alterations, such as reduced volumes, in the genu of the corpus callosum [34].

This study has the following three limitations: first, the sample size was small, which may have led to type II errors in the statistical analysis. Second, the patients with depression were not divided into subgroups (minor to severe), which may have influenced the discrimination results to some extent. Third, only 2 feature selection approaches were chosen for the comparison in the SVM classifier.

## Conclusion

Depression diagnosis has been a complex research problem for a long time, and a standard for diagnosing depression symptoms is currently unavailable. Thus, a quick, objective, and inexpensive assessment tool with a high accuracy for effective intervention strategies is necessary. In this study, we investigated the EEG features associated with depression, including relative frequency power, alpha interhemispheric asymmetry, left–right coherence, strength, CC, L, SampEn, MSE, and DFA, and compared the performance of the following two selection approaches, Student’s *t*-test and AUC, in the SVM classifier. The *t*-test selection demonstrated the highest performance in the eye-open and eye-closed conditions—and their accuracy, sensitivity, and specificity were 96.66%, 95.93%, and 97.550% in the eye-open condition and 91.33%, 90.59%, and 91.81% in the eye-closed condition, respectively. Furthermore, for the comparisons of features in the two selection approaches, the most influential features were relative frequency power and left–right coherence.

Although our current results indicate that the SVM classifier may not be robust enough for clinical application, the current method warrants further exploration because of its simplicity, objective, and efficiency. Our results may also be useful in advancing research in the early identification of individuals at the early stages of MDD (i.e., patients with mild MDD symptoms) and can contribute to the development of a wider variety of clinical applications in the future.
